# Whole-genome analysis of a novel Pandoraea sputorum lineage causing high-mortality bloodstream infections

**DOI:** 10.1099/mgen.0.001663

**Published:** 2026-06-02

**Authors:** Hua Wu, Lin Liu, Shijia Li, Pei Zhang, Ziling Xing, Qian Huang, Dai Kuang, Wei Liu, Chong Chen, Qianfeng Xia

**Affiliations:** 1 Department of Clinical Laboratory, Hainan Women and Children Medical Center, Hainan Medical University, Haikou 570312, Hainan, PR China; 2 NHC Key Laboratory of Tropical Disease Control, School of Life Sciences and Medical Technology, Hainan Medical University, Haikou 571199, Hainan, PR China; 3 Department of Clinical Laboratory, Hainan General Hospital, Hainan Medical University Hainan Hospital, Haikou 570311, Hainan, PR China; 4 Department of Clinical Laboratory, Hainan Second People’s Hospital, Wuzhishan 572200, Hainan, PR China

**Keywords:** antimicrobial resistance, bloodstream infection, molecular epidemiology, *Pandoraea sputorum*, phylogenomics, whole-genome sequencing

## Abstract

**Background.** *Pandoraea sputorum* is an emerging multidrug-resistant pathogen primarily associated with cystic fibrosis. Bloodstream infections (BSIs) are rare but linked to high mortality. The genomic characteristics and evolutionary history of *P. sputorum* lineages causing BSIs remain poorly understood.

**Methods.** We performed whole-genome sequencing on 18 *P*. *sputorum* isolates recovered from patients with BSIs in China between June 2022 and March 2024. Phylogenetic relationships were determined by core-genome SNP analysis, and the pan-genome was characterized using Roary. Antimicrobial resistance and virulence genes were identified using the CARD and VFDB databases. Evolutionary timescales were estimated using Bayesian molecular clock analysis.

**Results.** The case fatality rate among patients was 27.8% (5 out of 18). Common clinical manifestations included fever (83.3%, 15 out of 18), productive cough (77.8%, 14 out of 18) and dyspnoea (61.1%, 11 out of 18). All isolates formed a distinct monophyletic lineage, genetically distant (>2,000 SNPs) from known *P. sputorum* strains but with minimal intra-lineage diversity (15–98 SNPs), indicating recent clonal expansion. The pan-genome was highly conserved, with core genes comprising 94.6% of the gene repertoire. All strains harboured core genome-encoded *ceoB* (efflux pump) and *OXA-155* (β-lactamase) genes, conferring resistance to multiple antimicrobials but remaining susceptible to imipenem. Bayesian analysis estimated the origin of this lineage in 2004 (95% highest posterior density: 2003.2–2005.8), with major diversification occurring between 2007 and 2018.

**Conclusion.** We identified a novel, recently emerged and highly clonal lineage of *P. sputorum* associated with high-mortality BSIs. Its stable reservoir of core genome-encoded resistance mechanisms poses a significant therapeutic challenge. This lineage represents an emerging multidrug-resistant threat to public health, necessitating enhanced surveillance and infection control measures.

**Impact Statement**

*Pandoraea sputorum* is an emerging multidrug-resistant pathogen capable of causing fatal infections. This study identifies a novel, highly clonal lineage of *P. sputorum* responsible for high-mortality bloodstream infections in non-cystic fibrosis patients in China. Our findings reveal its recent evolutionary origin, stable core-genome-encoded resistance mechanisms and clonal expansion, highlighting this lineage as a significant emerging threat to public health that warrants enhanced surveillance and control measures.

## Data Summary

The whole-genome sequencing data generated in this study have been deposited in the NCBI database under BioProject accession number PRJNA1320643. Table S1 lists the individual BioSample and GenBank assembly accessions for each of the 18 *Pandoraea sputorum* strains studied.

## Data Availability

All *Pandoraea sputorum* strains used in this study were clinical isolates obtained from patients with bloodstream infections. The strains are currently preserved in the Department of Clinical Laboratory, Hainan General Hospital, and are available for research purposes from the corresponding author upon reasonable request.

## Introduction

*Pandoraea sputorum* is a non-fermentative Gram-negative bacillus that was initially regarded as an emerging pathogen colonizing the respiratory tract of patients with cystic fibrosis (CF), where it has been associated with progressive decline in lung function [1,3]. In recent years, however, the clinical spectrum of *P. sputorum* has expanded beyond CF, with invasive infections such as pneumonia, skin and soft tissue infections and bacteraemia being increasingly reported in non-CF individuals. Cases have been documented in several countries, including Spain, Australia, France and the USA [4]. Recent systematic reviews have reported an overall mortality rate of ~30.23% for *Pandoraea* infections [5]. In addition, *P. sputorum* bloodstream infections are exceedingly rare, with only sporadic cases described since the first report in an organ transplant recipient in 2019 [6], yet available evidence suggests poor outcomes [7].

In terms of antimicrobial susceptibility, *P. sputorum* exhibits extensive resistance to multiple drug classes, generally remaining susceptible only to tetracyclines, imipenem and trimethoprim-sulfamethoxazole. Moreover, it shows a distinctive carbapenem resistance profile, being susceptible to imipenem but resistant to meropenem, a phenomenon potentially associated with OXA-type β-lactamases and multidrug efflux pumps [8].

*P. sputorum* was reclassified from the genus *Burkholderia* in 2000, but its close phenotypic similarity to *Burkholderia* and *Ralstonia* makes accurate identification challenging with conventional biochemical assays [1,3]. The combined application of 16S rRNA gene sequencing and MALDI-TOF MS can significantly improve diagnostic accuracy [4,9,12]. Nevertheless, current research has primarily focused on sporadic case reports, and systematic genomic investigations of bloodstream infection-associated *P. sputorum* strains remain lacking, particularly regarding their molecular epidemiology, resistance mechanisms and phylogenetic characteristics.

To date, only a limited number of *P. sputorum* genome sequences have been reported, primarily from sporadic CF cases or environmental sources. The recent analysis of 12 clinical isolates from Wuhan represents one of the few genomic studies focused on this species. Our addition of 18 bloodstream infection genomes therefore significantly expands the genomic repertoire available for understanding the pathogen’s evolution and clinical adaptation.

Therefore, we performed whole-genome sequencing of 18 *P*. *sputorum* bloodstream isolates collected between 2022 and 2024 to elucidate their genomic architecture, resistance mechanisms and clonal dynamics, thereby providing insights to support diagnosis, treatment and infection control.

## Methods

### Clinical strain collection and basic characteristics

Clinical data were extracted from electronic medical records by two independent investigators, covering demographic variables, hospitalization details, clinical manifestations, diagnostic findings, antimicrobial treatment history, comorbidities and outcomes. Accuracy was ensured through dual verification, with discrepancies resolved by consensus. Bloodstream isolates of *Pandoraea* species were deemed clinically significant following independent evaluation by two infectious disease specialists.

Positive blood culture broths were subcultured onto sheep blood agar and incubated aerobically at 37 °C for 24–48 h. Colonies were assessed for morphological and Gram staining, and Gram-negative isolates were further subcultured onto blood agar and MacConkey agar plates (Autobio Diagnostics Co., Ltd., Zhengzhou, China) for preliminary identification.

Antimicrobial susceptibility testing was performed using the broth microdilution method according to CLSI guidelines. MICs were determined for 12 antimicrobial agents: ceftazidime, meropenem, imipenem, piperacillin/tazobactam, gentamicin, ciprofloxacin, doxycycline, tigecycline, trimethoprim-sulfamethoxazole, cefoperazone/sulbactam and chloramphenicol. *Pseudomonas aeruginosa* ATCC 27853 and *Escherichia coli* ATCC 25922 served as quality control strains. MIC endpoints were recorded after 18–24 h incubation at 37 °C.

To minimize and monitor potential laboratory contamination, samples were processed independently across different time points and clinical departments. DNA extraction and library preparation were performed in separate batches with negative controls (sterile water) included in each batch. No amplification was observed in negative controls. These procedures were implemented to rigorously exclude and monitor the possibility of laboratory contamination.

### Genome sequencing and strain identification

Genomic DNA was extracted from pure bacterial cultures using the DNeasy UltraClean Microbial Kit (Qiagen, Hilden, Germany) according to the manufacturer’s instructions. DNA concentration and purity were assessed using a NanoDrop 2000 spectrophotometer (Thermo Fisher Scientific, USA), and integrity was confirmed by 1.0% agarose gel electrophoresis.

Whole-genome sequencing was performed on the Illumina MiSeq platform (Illumina, San Diego, CA, USA). Paired-end sequencing libraries were prepared using the Nextera XT DNA Library Preparation Kit (Illumina) following standard protocols, which included DNA fragmentation, end repair, adapter ligation and library amplification. The resulting libraries were quantified using a Qubit 4.0 Fluorometer (Thermo Fisher Scientific) and sequenced with a 2×150 bp paired-end strategy.

Raw sequencing reads were *de novo* assembled using SPAdes assembler (v3.15.3) with default parameters. The resulting genome assemblies were automatically annotated using Prokka software [13,14]. Preliminary strain identification was performed by comparing the assembled 16S rRNA gene sequences against the NCBI nucleotide database using BLASTn. The quality of genome assemblies was assessed based on metrics including genome size, GC content, N50 and number of contigs.

### Phylogenetic and average nucleotide identity analysis

High-quality genome sequences of all *Pandoraea* species were downloaded from public databases, and their 16S rRNA gene sequences were extracted for phylogenetic analysis with the study strains. Maximum-likelihood phylogenetic trees were constructed using appropriate phylogenetic software with bootstrap resampling to assess the taxonomic classification of the isolates [15,16].

Average nucleotide identity (ANI) analysis was performed between a representative strain from this study and high-quality genomes of *Pandoraea* species. Genome-wide ANI values were calculated, with the established threshold of ≥95% ANI used for species-level identification [17].

### Core genome phylogenetic analysis

Core genome-based phylogenetic analysis was conducted using the study strains and known *P. sputorum* reference strains. Core genome alignment was performed using Parsnp software to identify SNPs, and genetic distances between strains were calculated based on SNP differences. Phylogenetic trees were constructed to determine the evolutionary relationships among the strains [18].

### Pan-genome analysis

Pan-genome analysis of the *P. sputorum* clade was performed using Roary software. Genes were classified according to their distribution frequency across genomes, and the total pan-genome size and core genome complement were determined [19].

### Functional gene annotation

Functional annotation of the pan-genome was conducted using ABRicate software for both virulence factor and antimicrobial resistance gene identification. Virulence factor annotation was performed against the Virulence Factor Database (VFDB), while antimicrobial resistance genes were identified using the Comprehensive Antibiotic Resistance Database (CARD). The number of predicted virulence and resistance genes was quantified, and their distribution within the core genome was analysed [20,21].

### Molecular clock analysis

Prior to molecular clock analysis, temporal signal was assessed using root-to-tip regression analysis in TempEst v1.5.3 to evaluate the correlation between genetic distance and sampling time. Molecular clock analysis of the novel *P. sputorum* clade was performed using BEAST software based on core genome sequence alignments generated by Parsnp. The optimal nucleotide substitution model was determined using jModelTest software. Multiple combinations of clock and tree models were tested, including strict and uncorrelated relaxed clock models, along with constant size, exponential growth, Bayesian skyline and Bayesian skygrid demographic models [22,24]. Markov chain Monte Carlo analyses were run for 100,000,000 generations. The optimal model combination was selected through model comparison, and maximum clade credibility (MCC) trees were constructed to infer the evolutionary timeline and estimate the time to most recent common ancestor (tMRCA) of the clade.

## Results

### Clinical characteristics and treatment outcomes

Between June 2022 and March 2024, *P. sputorum* strains were isolated from 18 patients with bloodstream infections. *P. sputorum* was the sole pathogen detected in 16 out of 18 patients. Two patients had polymicrobial bacteremia: WP24 co-isolated with *Sphingomonas paucimobilis*, and WP46 with *Ralstonia pickettii*. The median age was 62 years (range 34–90 years), with 10 males (55.6%) and 8 females (44.4%). Farmers comprised the majority (13 cases, 72.2%), followed by retired individuals (4 cases, 22.2%). Underlying diseases were present in 13 patients (72.2%), with hypertension being the most common (8 cases, 44.4%), followed by stroke (4 cases, 22.2%) and diabetes mellitus (2 cases, 11.1%).

Fever was the most common clinical manifestation (15 cases, 83.3%), followed by cough with sputum (14 cases, 77.8%) and chest tightness with dyspnoea (11 cases, 61.1%). Altered consciousness occurred in 4 patients (22.2%), and seizures in 3 patients (16.7%). Severe complications included respiratory failure in 4 cases (22.2%), septic shock in 1 case (5.6%), heart failure in 2 cases (11.1%) and multiple organ dysfunction in 3 cases (16.7%).

The overall case fatality rate was 27.8% (5 out of 18), with altered consciousness, respiratory failure and multiple organ dysfunction being significantly more common in the non-survivor group. The median time to positive blood culture was 3 days (Table 1). The heterogeneity in clinical presentations, outcomes and the presence of polymicrobial bacteraemia in two cases strongly support the clinical relevance and independent origin of each *P. sputorum* isolate, arguing against a common source of laboratory contamination.

**Table 1.** Clinical characteristics and treatment outcomes of patients with *P. sputorum* bloodstream infections

Characteristic	All patients (*n*=18)	Survivors (*n*=13)	Non-survivors (*n*=5)
**Demographics**			
Age, median (IQR), years	62 (54–73)	62 (51–68)	58 (55–75)
Age ≥65 years, n (%)	9 (50.0)	6 (46.2)	3 (60.0)
Male gender, n (%)	10 (55.6)	7 (53.8)	3 (60.0)
**Occupation, n (%)**			
Farmer	13 (72.2)	10 (76.9)	3 (60.0)
Retired	4 (22.2)	2 (15.4)	2 (40.0)
Other	1 (5.6)	1 (7.7)	0 (0.0)
**Underlying diseases, n (%)**			
Any underlying disease	13 (72.2)	9 (69.2)	4 (80.0)
Hypertension	8 (44.4)	5 (38.5)	3 (60.0)
Stroke	4 (22.2)	2 (15.4)	2 (40.0)
Diabetes mellitus	2 (11.1)	2 (15.4)	0 (0.0)
Liver cirrhosis	1 (5.6)	0 (0.0)	1 (20.0)
**Clinical presentations, n (%)**			
Fever	15 (83.3)	11 (84.6)	4 (80.0)
Cough with sputum	14 (77.8)	10 (76.9)	4 (80.0)
Chest tightness/dyspnoea	11 (61.1)	8 (61.5)	3 (60.0)
Altered consciousness	4 (22.2)	1 (7.7)	3 (60.0)
Seizures	3 (16.7)	2 (15.4)	1 (20.0)
Abdominal pain/diarrhoea	3 (16.7)	2 (15.4)	1 (20.0)
**Severe complications, n (%)**			
Respiratory failure	4 (22.2)	1 (7.7)	3 (60.0)
Septic shock	1 (5.6)	1 (7.7)	0 (0.0)
Heart failure	2 (11.1)	1 (7.7)	1 (20.0)
Multiple organ dysfunction	3 (16.7)	0 (0.0)	3 (60.0)
**Primary infection sites, n (%)**			
Pulmonary	12 (66.7)	9 (69.2)	3 (60.0)
Gastrointestinal	2 (11.1)	2 (15.4)	0 (0.0)
Central nervous system	4 (22.2)	2 (15.4)	2 (40.0)
**Antibiotic treatment, n (%)**			
β-Lactam monotherapy	9 (50.0)	7 (53.8)	2 (40.0)
Fluoroquinolone monotherapy	1 (5.6)	1 (7.7)	0 (0.0)
Combination therapy	7 (38.9)	5 (38.5)	2 (40.0)
No antibiotic treatment	1 (5.6)	0 (0.0)	1 (20.0)
**Blood culture positivity**			
Time to detection, median (IQR), days	3 (1–4)	3 (2–4)	2 (1–3)
**Outcomes**			
Case fatality rate, n (%)	5 (27.8)	–	–

Data are presented as number of cases (percentage) for categorical variables and median (interquartile range) for continuous variables. ‘–’ indicates that the item is not applicable.

IQR, interquartile range.

### Antimicrobial susceptibility profiles

MIC determinations were performed for 12 antibiotics against 13 *P. sputorum* isolates. MIC values for ceftazidime, meropenem and gentamicin reached or exceeded the testing limits (≧128 µg ml^−1^, ≧16 µg ml^−1^ and ≧16 µg ml^−1^, respectively). The MIC for piperacillin/tazobactam was ≧16/4 µg ml^−1^, with only one strain showing an MIC of 8/4 µg ml^−1^. Imipenem demonstrated an MIC of 1 µg ml^−1^, with 11 of 13 strains showing MIC values≤1 µg ml^−1^, in contrast to meropenem, where all strains had MIC values ≧16 µg ml^−1^. The MIC values for doxycycline, tigecycline and trimethoprim-sulfamethoxazole were 0.5 µg ml^−1^, 1 µg ml^−1^ and 0.25/4.75 µg ml^−1^, respectively. Cefoperazone/sulbactam showed an MIC of 4/2 µg ml^−1^, ciprofloxacin 2 µg ml^−1^ and chloramphenicol 16 µg ml^−1^. The low MIC values of doxycycline, tigecycline, trimethoprim-sulfamethoxazole and imipenem suggest potential clinical therapeutic utility.

### Phylogenetic analysis and species identification

**Fig. 1.**
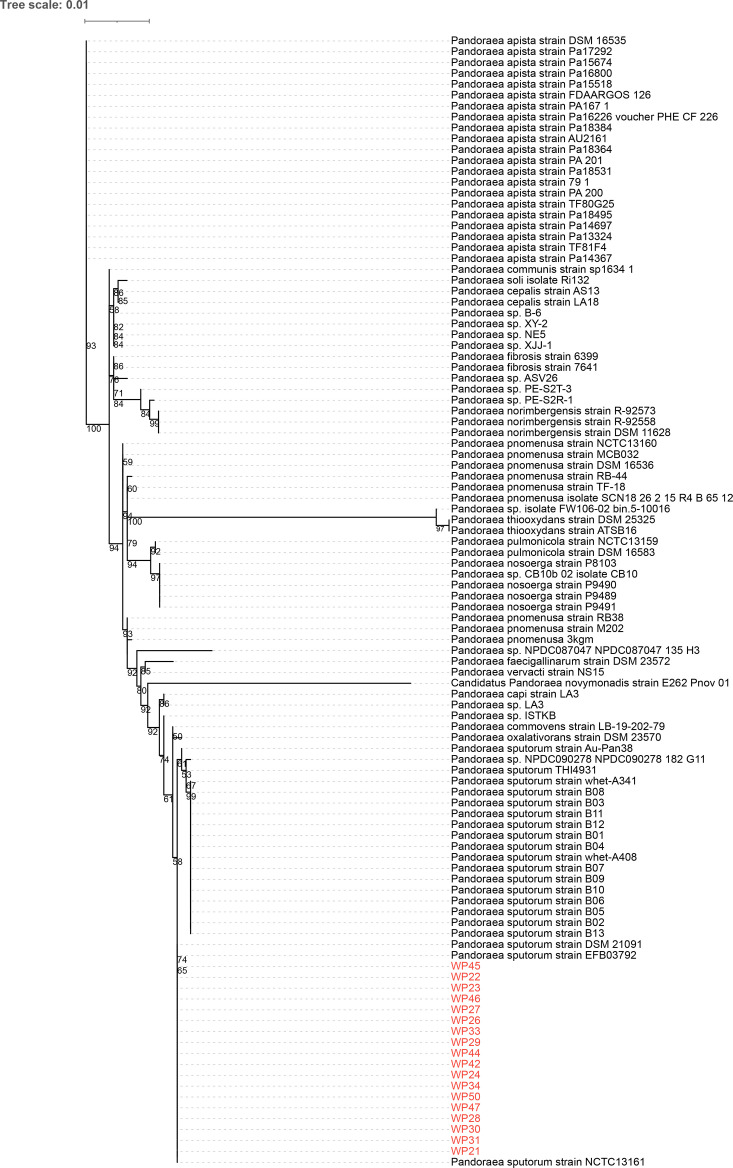
Maximum-likelihood phylogenetic tree of *Pandoraea* species based on 16S rRNA gene sequences. Bootstrap values (based on 1,000 replicates) are shown at key nodes. All 18 clinical strains from this study (highlighted in red) cluster within the *P. sputorum* clade. The scale bar represents nucleotide substitutions per site.

Phylogenetic analysis based on 16S rRNA gene sequences revealed the evolutionary relationships among different species within the genus *Pandoraea*. The maximum-likelihood tree demonstrated clear species-level clustering, with distinct phylogenetic branches formed by *Pandoraea apista*, *Pandoraea fibrosis*, *Pandoraea norimbergensis*, *Pandoraea pnomenusa*, *Pandoraea pulmonicola*, *Pandoraea nosoerga* and other recognized species within the genus. All 18 strains from this study (WP21, WP24, WP26, WP27, WP28, WP29, WP30, WP31, WP42, WP44, WP45, WP46, WP47, WP49, WP50 and others) clustered exclusively with known *P. sputorum* reference strains, forming a well-supported monophyletic group that was clearly separated from other *Pandoraea* species (Fig. 1). This 16S rRNA gene-based phylogeny provided preliminary phylogenetic placement within the genus. For definitive species identification and all subsequent high-resolution genomic analyses (including ANI and core-genome SNP phylogeny), we relied on whole-genome sequence data.

**Fig. 2.**
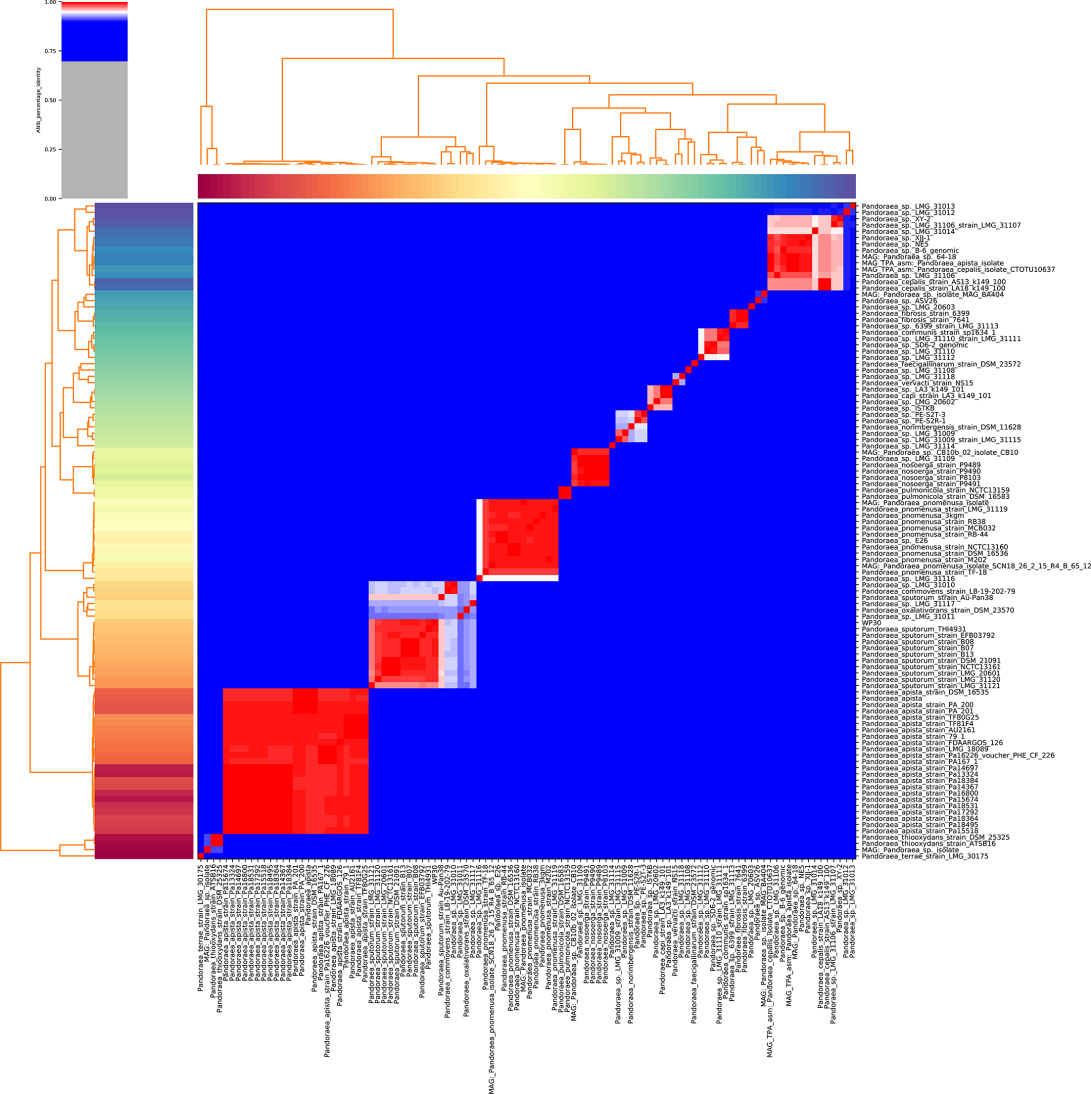
ANI heatmap between a representative study strain (WP30) and reference genomes of various *Pandoraea* species. The dashed line indicates the 95% ANI threshold for species boundary.

To further validate species-level identification, ANI analysis was performed to assess genome-wide similarity relationships among *Pandoraea* strains. The representative strain WP30 exhibited high genomic similarity with all known *P. sputorum* reference strains, with ANI values consistently exceeding the established 95% threshold for species delineation (Fig. 2).

### Core genome phylogenetic analysis

**Fig. 3.**
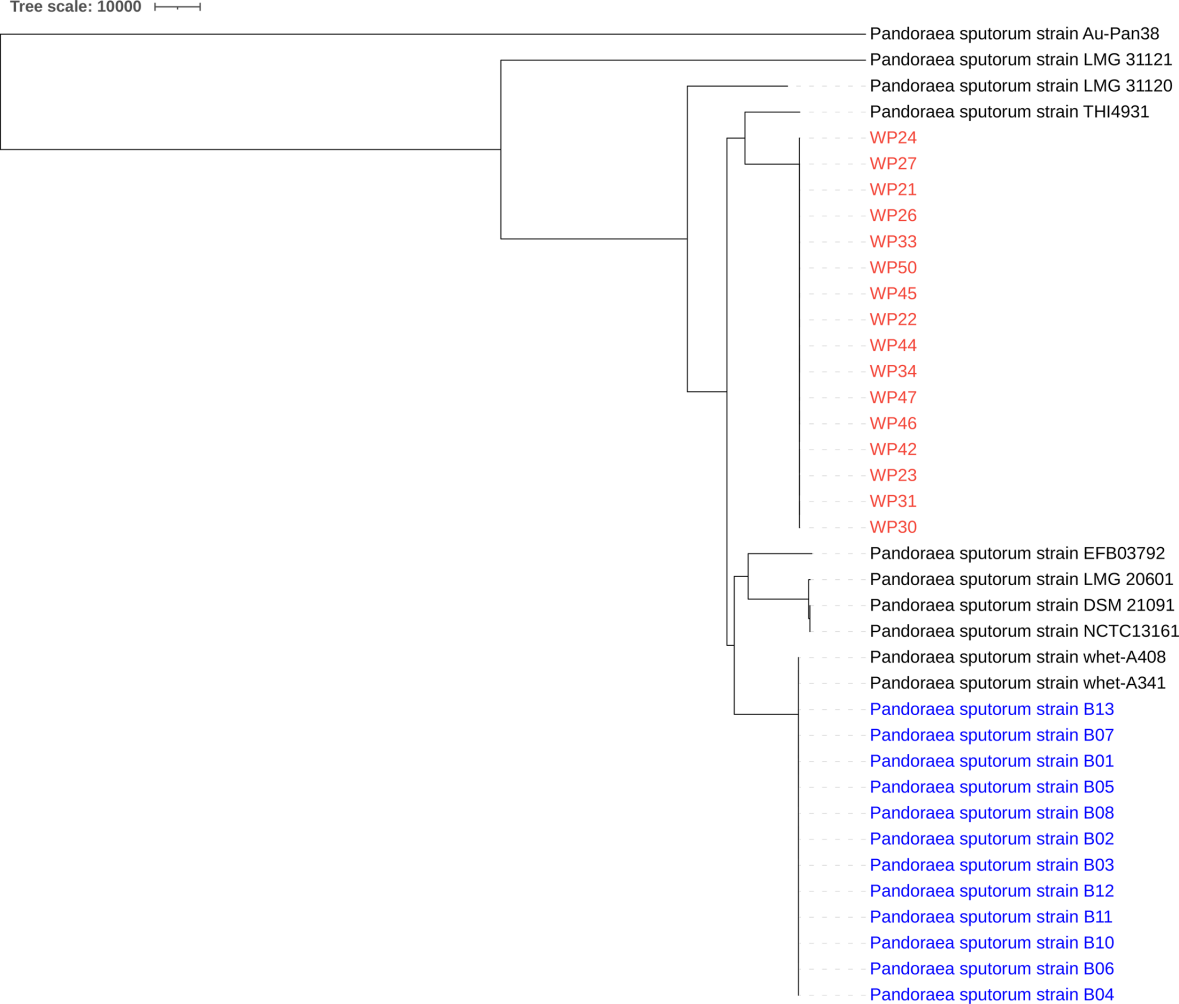
Core genome SNP-based maximum-likelihood phylogeny of *P. sputorum* strains. The 18 study strains form a distinct, monophyletic clade (highlighted in red). The recently reported 12 isolates from Wuhan, China [25], form a separate monophyletic clade (highlighted in blue). Both clades are separated from known reference strains by >2,000 SNPs. Reference strains are labelled with their accession numbers and source countries.

Core genome phylogenetic analysis revealed the evolutionary relationships within the *P. sputorum* species complex. The maximum-likelihood tree constructed from core genome SNPs demonstrated that all 18 study strains (WP21, WP22, WP23, WP24, WP26, WP27, WP28, WP29, WP30, WP31, WP33, WP34, WP42, WP44, WP45, WP46, WP47 and WP50) formed a distinct monophyletic clade that was clearly separated from known *P. sputorum* reference strains (Fig. 3). The recently reported 12 *P. sputorum* isolates from Wuhan, China [25], formed a separate, monophyletic clade distinct from our lineage. The intra-clade genetic diversity among the 18 study strains was remarkably low, with pairwise SNP differences consistently below 100 nucleotides (range: 15–98 SNPs), indicating recent divergence from a common ancestor and suggesting clonal expansion or limited genetic recombination within this lineage. Conversely, the inter-clade genetic distance between the novel lineage and previously characterized *P. sputorum* strains was substantial, exceeding 2,000 SNPs in all pairwise comparisons (range: 2,156–2,847 SNPs). The recently reported 12 *P. sputorum* genomes from Wuhan [25] were included in this core-genome phylogeny for comparative analysis. The core genome alignment used for SNP calling comprised 4,718,624 bp, representing conserved genomic regions across all studied and reference strains.

### Pan-genome architecture and gene content analysis

**Fig. 4.**
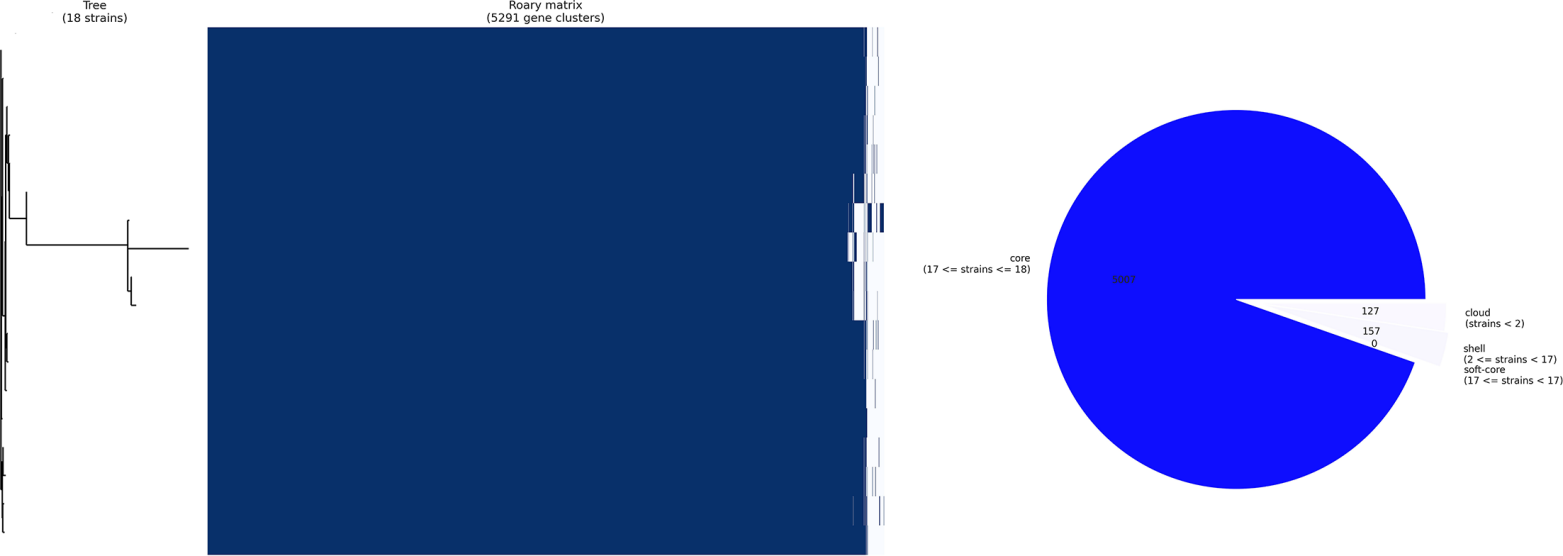
Pan-genome structure of the novel *P. sputorum* lineage. (Left) Gene presence (blue) and absence (white) matrix across the 18 strains with a phylogenetic tree based on gene content. (Right) Pie chart illustrating the pan-genome composition, showing a highly conserved core genome (94.6%).

Pan-genome analysis of the 18 *P. sputorum* strains revealed a highly conserved genomic architecture characteristic of a closed pan-genome. The complete pan-genome comprised 5,291 gene clusters, with the core genome accounting for 5,007 genes (94.6% of the total pan-genome), demonstrating remarkable genetic conservation within this lineage (Fig. 4). The accessory genome was limited, consisting of 127 cloud genes (present in <15% of strains), 157 shell genes (present in 15–95% of strains) and no soft-core genes (95–99% presence), indicating minimal horizontal gene transfer and limited genomic plasticity among the study strains.

The Roary matrix visualization demonstrated uniform gene presence patterns across all 18 strains, with the vast majority of genes showing universal distribution (Fig. 4, left panel). The phylogenetic tree based on gene presence/absence patterns was congruent with the core genome SNP-based phylogeny, reinforcing the monophyletic nature of this *P. sputorum* lineage. The pie chart representation clearly illustrated the predominance of core genes, with accessory genes comprising only 5.4% of the total gene repertoire (Fig. 4, right panel). The high core-to-pan genome ratio (0.946) and the minimal accessory genome content suggest that this *P. sputorum* lineage has undergone recent clonal expansion with limited gene acquisition or loss events.

### Virulence factor and antimicrobial resistance gene analysis

Screening against the VFDB identified a *cheW* gene (VF0430), encoding a chemotaxis signalling protein involved in flagellar motility regulation (Table 2).

**Table 2.** Virulence factors and antimicrobial resistance genes identified in the *P. sputorum* lineage

Gene	Database	Product	Function/resistance	Coverage (%)	Identity (%)	Genome location
*cheW*	VFDB	Chemotaxis protein CheW	Flagellar motility	82.39	85.29	Core genome
*ceoB*	CARD	CeoAB-OpcM efflux pump component	Aminoglycoside; fluoroquinolone	99.68	80.38	Core genome
*OXA-155*	CARD	OXA-155 β-lactamase	Carbapenem; penam	100.00	99.55	Core genome

All genes were present in all 18 strains (18 out of 18) and are classified as core genome elements.

CARD, Comprehensive Antibiotic Resistance Database; VFDB, Virulence Factor Database.

Antimicrobial resistance profiling using the CARD revealed two resistance genes, both classified as core genome components with 100% prevalence across the strain collection (Table 1). The *ceoB* gene (accession U97042.1) encodes a cytoplasmic membrane protein component of the CeoAB-OpcM tripartite efflux system, conferring phenotypic resistance to aminoglycosides and fluoroquinolones through active drug extrusion. Sequence analysis demonstrated 80.38% identity with 99.68% coverage to the reference sequence. The second resistance determinant, *OXA-155* (accession KP771983.1), represents a class D β-lactamase with 99.55% amino acid identity and complete sequence coverage (100%). This enzyme confers hydrolytic activity against carbapenem and penam antibiotics according to the Lahey β-lactamase classification system. The exclusive localization of resistance genes within the core genome indicates stable vertical inheritance rather than horizontal acquisition events, suggesting intrinsic resistance mechanisms characteristic of this phylogenetic lineage.

### Temporal evolution and molecular clock analysis

**Fig. 5.**
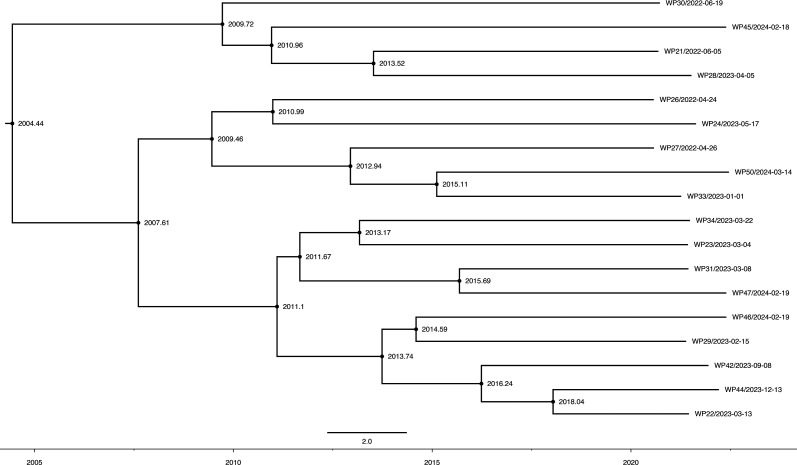
Evolutionary timeline of the novel *P. sputorum* lineage. The MCC tree was inferred using Bayesian molecular clock analysis. Node bars represent the 95% HPD intervals. The timescale of the analysis is shown in years.

Root-to-tip regression analysis revealed a positive temporal signal (R²=0.18; Fig. S1, available in the online Supplementary Material), supporting the subsequent application of Bayesian molecular clock models. The MCC tree, constructed using the optimal GTR+uncorrelated relaxed clock+exponential growth model combination, estimated the tMRCA of this lineage at 2004.44 [95% highest posterior density (HPD): 2003.2–2005.8], indicating a relatively recent evolutionary origin (Fig. 5).

The phylochronological reconstruction demonstrated that the majority of strain diversification occurred between 2007 and 2018, with multiple independent lineage splits documented throughout this period. Early divergence events were observed around 2007.61, leading to the separation of two major sub-lineages. Subsequent radiation events occurred primarily during 2009–2016, with notable branching points at 2009.46, 2009.72, 2010.96, 2010.99, 2011.1, 2011.67, 2012.94, 2013.17, 2013.52 and 2013.74, suggesting periods of rapid diversification within specific temporal windows.

## Discussion

The clinical significance of *P. sputorum* bloodstream infections has gained considerable attention, particularly given their associated mortality burden. A recent systematic review by Zarogiannis *et al.* analysed 43 *Pandoraea* infections and reported an overall mortality rate of 30.23% [5]. Our observed mortality rate of 27.8% is highly consistent with this data, further confirming the high pathogenicity of *P. sputorum* as an emerging pathogen. Notably, our study population consisted predominantly of farmers (72.2%) and exclusively of non-CF patients, contrasting with the traditional association of *Pandoraea* infections with CF [5]. This pattern suggests environmental exposure as a potential source of infection and broadens the recognized epidemiological spectrum of the pathogen. Furthermore, *Pandoraea* species have been shown to induce IL-6 and IL-8 production, eliciting strong pro-inflammatory responses [26] that may underlie the clinical manifestations of altered consciousness, respiratory failure and multiple organ dysfunction observed in affected patients. The first reported *P. sputorum* bloodstream infection occurred in a liver transplant recipient in 2019, characterized by severe immunosuppression [6]. Subsequent sporadic reports, including a non-CF patient who developed sepsis following polytrauma but ultimately recovered [7], further underscore the clinical complexity and heterogeneity of disease caused by this organism.

Integration of recently published *P. sputorum* genomes from Wuhan [25] revealed that our lineage and the Wuhan strains form two distinct, monophyletic clades within the species. This geographical substructure, independently identified by separate laboratories, argues against laboratory contamination as an explanation for the low diversity observed in our study and highlights the emerging genetic diversity of clinical *P. sputorum* populations in China.

Beyond its pathogenic potential, *P. sputorum* poses substantial therapeutic challenges. In Argentina, isolates from CF patients were susceptible only to imipenem and trimethoprim-sulfamethoxazole, yet bacterial eradication was not achieved despite clinical improvement [4]. Similarly, a long-term Spanish study revealed the organism’s ability to persist in the respiratory tract, with chronic infection between 2005 and 2008 associated with progressive pulmonary function decline, whereas re-isolation in 2010 responded favourably to imipenem [27]. Further underscoring its clinical importance, a recent outbreak of *Pandoraea pneumonica* in Hainan, China, involving 30 patients with a mortality rate of 16.67% [28], demonstrates the emerging role of *Pandoraea* species as bloodstream pathogens in Asia, particularly in tropical regions that may favour environmental persistence.

Integration of recently published *P. sputorum* genomes from Wuhan [25] revealed that our lineage and the Wuhan strains form two distinct, monophyletic clades within the species. This geographical substructure, independently identified by separate laboratories, argues against laboratory contamination as an explanation for the low diversity observed in our study and highlights the emerging genetic diversity of clinical *P. sputorum* populations in China.

Furthermore, the likelihood of systematic laboratory contamination is minimized by several lines of evidence from this study itself. First, the isolates were collected over a 22-month period (June 2022 to March 2024) from patients with heterogeneous clinical features and outcomes, including two cases of polymicrobial bacteraemia, which is inconsistent with a single-point contamination event. Second, the observed low intra-lineage genomic diversity (15–98 SNPs) is characteristic of recent clonal expansion within a natural reservoir or host population, rather than the near-identity expected from repeated contamination from a common laboratory source. Together, the epidemiological, clinical and genomic data robustly refute contamination as a plausible explanation for our findings.

Through phylogenetic analysis of 18 bloodstream isolates, this study identified a distinct clade of *P. sputorum* that is significantly divergent from previously known strains, confirming the genetic heterogeneity within *P. sputorum*. This variability likely reflects adaptive divergence across different ecological niches [26,29] and suggests potential unique biological properties, akin to the genomovars observed in other opportunistic pathogens, such as the *Burkholderia cepacia* complex [30]. Bayesian molecular clock analysis provided critical insights into the temporal dynamics of this lineage. The most recent common ancestor is estimated to have emerged around 2004, followed by significant diversification between 2007 and 2018, exhibiting an exponential growth pattern. This timeline contrasts notably with the recent *Pandoraea commovens* outbreak in German intensive care units (2019–2021) [31], suggesting species-specific adaptive patterns in healthcare environments. The exponential expansion indicates selective advantages acquired in hospital settings, paralleling the clinical features observed in 24 critically ill non-CF patients during the German outbreak, all of whom shared common characteristics including ICU admission, invasive ventilation and prior antimicrobial therapy [31].

Functional analysis identified key virulence and resistance determinants underlying pathogenicity. The chemotaxis protein CheW represents a critical virulence factor, consistent with previous studies demonstrating *Pandoraea* species’ ability to invade A549 lung epithelial cells and induce inflammatory responses [26]. Chemotactic motility proves essential for bloodstream pathogen survival and dissemination within the circulatory system. The universal presence and high conservation of *cheW* across all isolates strongly suggest central importance in adaptive evolution. Additionally, we identified the characteristic carbapenem resistance pattern of *Pandoraea* species – imipenem susceptibility with meropenem resistance – previously documented in clinical studies [32]. The *ceoB*-encoded efflux pump further confers resistance to aminoglycosides and fluoroquinolones, creating complex multidrug-resistant phenotypes [33]. The oxacillinase variant, *OXA-1152*, in *P. sputorum* contributes to discrepancies in carbapenem resistance. Previous studies identified a previously uncharacterized oxacillinase gene, *bla*_OXA-1152_, in 12 *P. sputorum* strains from Wuhan, China. Deletion of the *bla*_OXA-1152_ gene reduced meropenem and imipenem MICs by 64-fold and 4-fold, respectively, while reintroduction of this gene into *P. sputorum* and *E. coli* DH5α restored antimicrobial resistance [25].

Despite the high genomic similarity among isolates (15–98 SNPs), clinical outcomes were heterogeneous (27.8% mortality). This genotype–phenotype discordance suggests that host-related factors, such as underlying comorbidities (e.g. hypertension and stroke), immune status, timing of appropriate therapy and infection parameters, may play a more critical role in determining disease severity than minor genomic variation. Additionally, the encoded OXA-155 β-lactamase may contribute to the distinctive carbapenem resistance profile (meropenem resistance/imipenem susceptibility), potentially through differential hydrolysis efficiency as suggested by kinetic parameters (kcat/Km) reported for related enzymes [33].

The molecular clock analysis should be interpreted with caution due to the relatively weak temporal signal (R²=0.18), likely a result of the short sampling timeframe and high clonality of the lineage. Nevertheless, the Bayesian analysis provided a plausible estimate for the recent origin and expansion of this clone, consistent with its epidemiological context.

In conclusion, this study systematically delineates the clinical features, antimicrobial resistance profiles and molecular epidemiological characteristics of *P. sputorum* bloodstream infections. It further reveals the highly conserved genetic architecture, evidence of clonal expansion and intrinsic resistance mechanisms of this pathogen. These findings confirm the severe pathogenicity and substantial mortality associated with *P. sputorum* in non-CF populations, thereby expanding current epidemiological understanding. Moreover, our study highlights its role as an emerging multidrug-resistant pathogen of public health concern and underscores the necessity for continuous surveillance and in-depth mechanistic investigations.

## Supplementary material

10.1099/mgen.0.001663 Uncited Table S1.

10.1099/mgen.0.001663 Uncited Fig. S1.

10.1099/mgen.0.001663 Uncited Fig. S2.

## Abbreviations

ANI: average nucleotide identity
BSIs: bloodstream infections
CARD: Comprehensive Antibiotic Resistance Database
CF: cystic fibrosis
HPD: highest posterior density
MCC: maximum clade credibility
VFDB: Virulence Factor Database
