# Supplementary material for: Whole-genome analysis of a novel Pandoraea sputorum lineage causing high-mortality bloodstream infections
Source: Microb Genom. 2026 Jun 2;12(6):001663. doi: 10.1099/mgen.0.001663 (PMC13229510; doi:10.1099/mgen.0.001663)
Supplement: Uncited Table S1. [file mgen-12-01663-s001.pdf]

Table S1: Strain information and NCBI accession numbers for the 18 *Pandoraea sputorum* isolates.

Strain ID	BioSample Accession	GenBank Assembly Accession
WP21	SAMN51154941	JBRFWQ000000000
WP22	SAMN51154942	JBRFWP000000000
WP23	SAMN51154943	JBRFWO000000000
WP24	SAMN51154944	JBRFWN000000000
WP26	SAMN51154945	JBRFWM000000000
WP27	SAMN51154946	JBRFWL000000000
WP28	SAMN51154947	JBRFWK000000000
WP29	SAMN51154948	JBRFWJ000000000
WP30	SAMN51154949	JBRFWI000000000
WP31	SAMN51154950	JBRFWH000000000
WP33	SAMN51154951	JBRFWG000000000
WP34	SAMN51154952	JBRFWF000000000
WP42	SAMN51154953	JBRFWE000000000
WP44	SAMN51154954	JBRFWD000000000
WP45	SAMN51154955	JBRFWC000000000
WP46	SAMN51154956	JBRFWB000000000
WP47	SAMN51154957	JBRFWA000000000
WP50	SAMN51154958	JBRFVZ000000000
