# Supplementary figures and images for: Whole-genome analysis of a novel Pandoraea sputorum lineage causing high-mortality bloodstream infections
Source: Microb Genom. 2026 Jun 2;12(6):001663. doi: 10.1099/mgen.0.001663 (PMC13229510; doi:10.1099/mgen.0.001663)

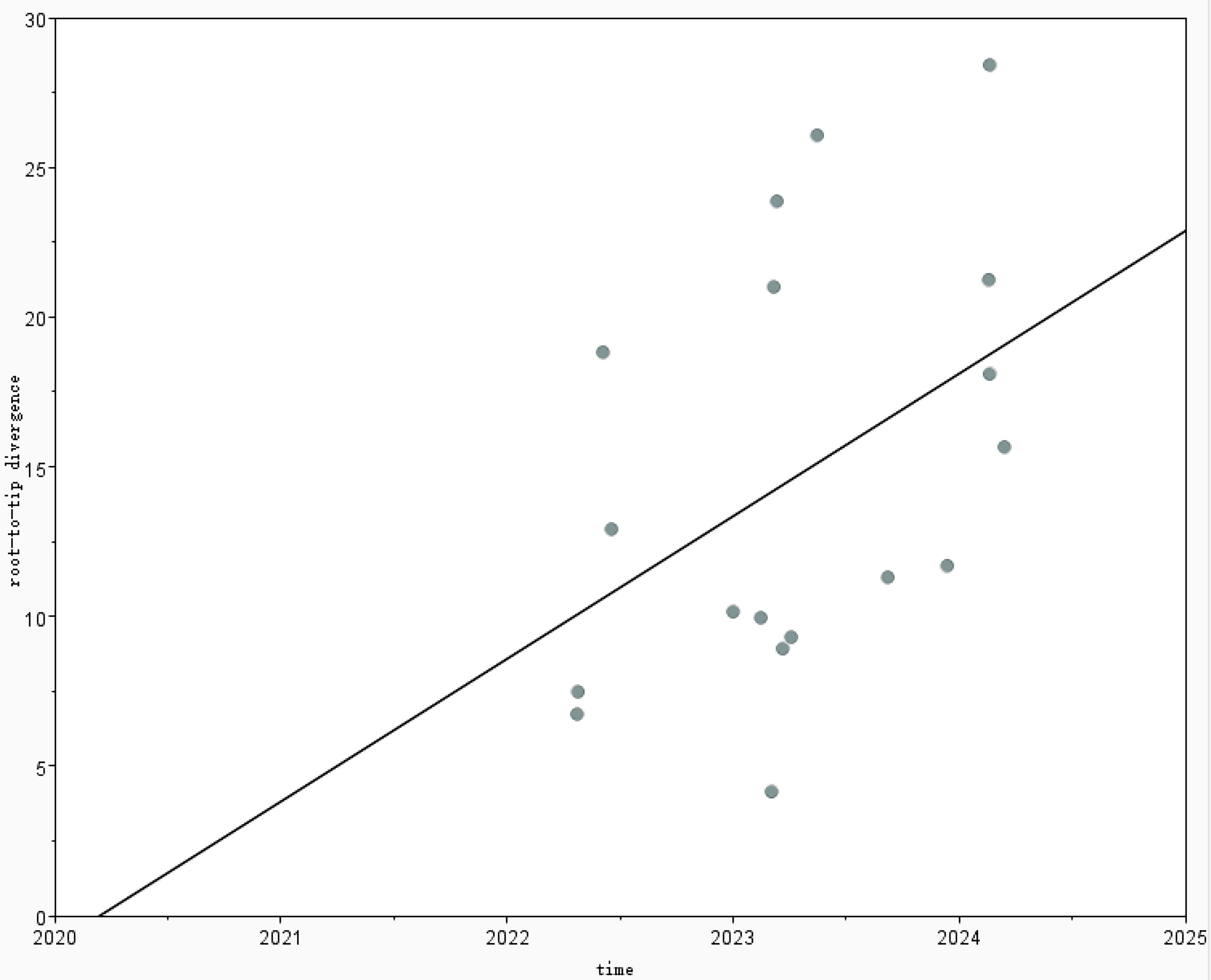

Supplement: Uncited Fig. S1. [file mgen-12-01663-s002.pdf]

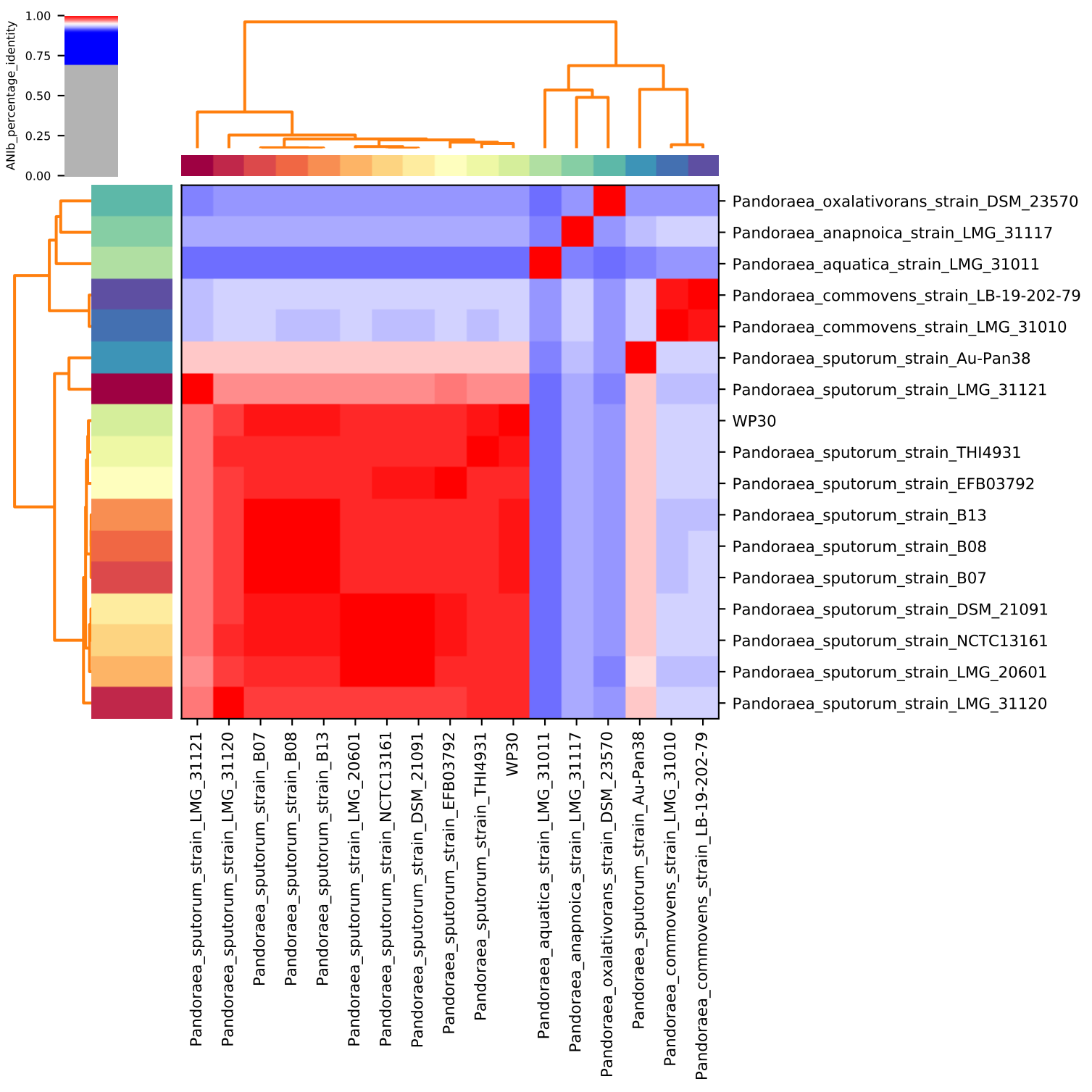

Supplement: Uncited Fig. S2. [file mgen-12-01663-s003.pdf]
